# The COnsensus-based Standards for the selection of health Measurement INstruments (COSMIN) and how to select an outcome measurement instrument

**DOI:** 10.1590/bjpt-rbf.2014.0143

**Published:** 2016-01-19

**Authors:** Lidwine B. Mokkink, Cecilia A. C. Prinsen, Lex M. Bouter, Henrica C. W. de Vet, Caroline B. Terwee

**Affiliations:** 1 Department of Epidemiology and Biostatistics, EMGO Institute for Health and Care Research, VU University Medical Center, Amsterdam, the Netherlands

**Keywords:** COSMIN, measurement properties, outcome measures, systematic reviews of instruments, outcome selection

## Abstract

**Background:**

COSMIN (COnsensus-based Standards for the selection of health Measurement INstruments) is an initiative of an international multidisciplinary team of researchers who aim to improve the selection of outcome measurement instruments both in research and in clinical practice by developing tools for selecting the most appropriate available instrument.

**Method:**

In this paper these tools are described, i.e. the COSMIN taxonomy and definition of measurement properties; the COSMIN checklist to evaluate the methodological quality of studies on measurement properties; a search filter for finding studies on measurement properties; a protocol for systematic reviews of outcome measurement instruments; a database of systematic reviews of outcome measurement instruments; and a guideline for selecting outcome measurement instruments for Core Outcome Sets in clinical trials. Currently, we are updating the COSMIN checklist, particularly the standards for content validity studies. Also new standards for studies using Item Response Theory methods will be developed. Additionally, in the future we want to develop standards for studies on the quality of non-patient reported outcome measures, such as clinician-reported outcomes and performance-based outcomes.

**Conclusions:**

In summary, we plea for more standardization in the use of outcome measurement instruments, for conducting high quality systematic reviews on measurement instruments in which the best available outcome measurement instrument is recommended, and for stopping the use of poor outcome measurement instruments.

## BULLET POINTS

- COSMIN aims to improve instrument selection in research and clinical practice.
- Description of COSMIN tools for selecting most appropriate instrument.
- Call for standardization in instrument use.
- Call for conducting high quality systematic reviews on instruments.
- Call for stopping the use of poor measurement instruments.

## Introduction

COSMIN (COnsensus-based Standards for the selection of health Measurement INstruments) is an initiative of an international multidisciplinary team of researchers with a background in epidemiology, psychometrics, qualitative research, and health care, who have expertise in the development and evaluation of outcome measurement instruments^1^. The COSMIN initiative aims to improve the selection of outcome measurement instruments both in research and in clinical practice by developing tools for selecting the most appropriate instrument. The COSMIN Steering Committee (see Appendix 1), founded in 2005, was inspired by a lack of clarity in the literature about terminology and definitions of measurement properties. Moreover, there exists an impressive amount of outcome measurement instruments and there are even many instruments measuring the same construct, developed for the same patient population, and still new ones are being developed. So researchers and clinicians have to choose the most suitable instrument for their application.

The process of selecting outcome measures for specific purposes is complex. Choices involve conceptual considerations, such as defining the construct and population; practical aspects, such as burden for patients and raters, and costs; and quality aspects assessed by nine different measurement properties clustered in the domains reliability, validity and responsiveness^2^. Selecting unsuitable or poor quality outcome measurement instruments may introduce bias in the conclusions of studies. This may lead to a waste of resources and be unethical because participating patients contribute little or nothing to the body of knowledge but still suffer from the burdens and risks of the study^3^.

An additional problem is that in systematic reviews of clinical trials the results reported cannot be compared and statistically pooled when different instruments are used to measure the same construct of interest in each study. Moreover, in clinical trials evaluating the benefits and harms of health care interventions, often a great variety of outcomes are reported. This makes it even more difficult to compare and combine results. This hampers the usefulness of clinical trial evidence to inform clinicians, at the cost of the best possible care for patients. Standardization in outcomes and outcome measurement instruments in specific areas of research is therefore highly warranted.

The COSMIN initiative wants to improve the selection of outcome measurement instruments by developing methodological guidelines based on consensus reached in a broad international panel of experts. The initial focus was on patient-reported outcome measures (PROMs). Therefore, the focus of this paper is only on PROMs.

First, some conceptual considerations concerning the selection of an outcome measurement instrument are explained. Next, the tools yielded by the COSMIN initiative will be described. Finally, we describe our future plans for research.

### Conceptual considerations when selecting outcome measurement instruments

It is important to understand the distinction between an outcome and an outcome measurement instrument. An outcome refers to the construct of interest. Since we talk about patient-reported outcomes, the outcome is often a phenomenon that cannot be observed directly, for example fatigue or health-related quality of life. The outcome chosen defines *what* is being measured. An outcome measurement instrument refers to *how* the outcome is being measured. It refers to the specific outcome measurement instrument. For example, the Neurological Fatigue Index for multiple sclerosis (NFI-MS)^4^ or the Skindex-29^5^ to measure quality of life in dermatology.

When selecting an outcome measurement instrument for research or clinical practice, first the outcome to be measured should be clearly defined. That is, one should define what to measure. For example, when measuring a broad construct such as health-related quality of life, it should be clarified which subdomains are relevant for the target population in the specific context of interest. Sometimes several definitions exist for an outcome. There are, for instance, multiple definitions for the construct 'disability'. The World Health Organization (WHO) defines 'disability' as a broad concept: 'problems an individual may experience in functioning, namely impairments, activity limitations and participation restrictions'^6^. Nagi^7^ defined disability more narrowly as 'a pattern of behaviour that evolves in situations of long-term or continued impairment that are associated with functioning limitations' (previously called 'handicap' in the International Classification of Functioning of the WHO^8^). Without explicitly defining or describing the intended outcome, people may have different ideas about it and interpret it differently.

Next, one has to choose a specific instrument. Often, for the same outcome multiple measurement instruments are available. To select the best available outcome measurement instrument the COSMIN initiative has yielded several tools.

Standardization of the selection of outcomes and outcome measurement instruments in specific areas of research will improve consistencies in reporting and decrease difficulties in comparing and combining the findings in systematic reviews and meta-analyses. This can be obtained by the development of Core Outcome Sets (COS). A COS is an agreed standardized set of outcomes that should be measured and reported, as a minimum, in all clinical trials in a specific disease or trial population (i.e. *what* to measure)^9^. Once the COS is defined, it is then important to achieve consensus on which outcome measurement instruments should be selected to measure the core outcomes, referring to Core Outcome Measurement Instruments (i.e *how* to measure)^10^. The existence or use of a core outcome set does not imply that outcomes in a particular trial should be restricted to those in the relevant core outcome set. Rather, there is an expectation that the core outcomes will be collected and reported, making it easier for the results of trials to be compared, contrasted and combined as appropriate; while researchers continue to explore other outcomes as well^11^.

### COSMIN tools

The COSMIN initiative has developed the following tools to help researchers and clinicians choosing the most appropriate outcome measurement instrument:

1. COSMIN taxonomy and definitions of measurement properties;
2. COSMIN checklist to evaluate the methodological quality of studies on measurement properties;
3. Search filter for finding studies on measurement properties;
4. Protocol for systematic reviews of outcome measurement instruments;
5. Database of systematic reviews of outcome measurement instruments;
6. Guideline for selecting outcome measurement instruments for outcomes included in a Core Outcome Set.

We performed an international Delphi study aiming to develop consensus-based standards for assessing the methodological quality of studies on measurement properties^1^^,^ 2 ^,^ 12 ^-^ 14. Results from this study were the COSMIN taxonomy and definitions, and the COSMIN checklist.

### COSMIN taxonomy and definitions

**Figure 1:**
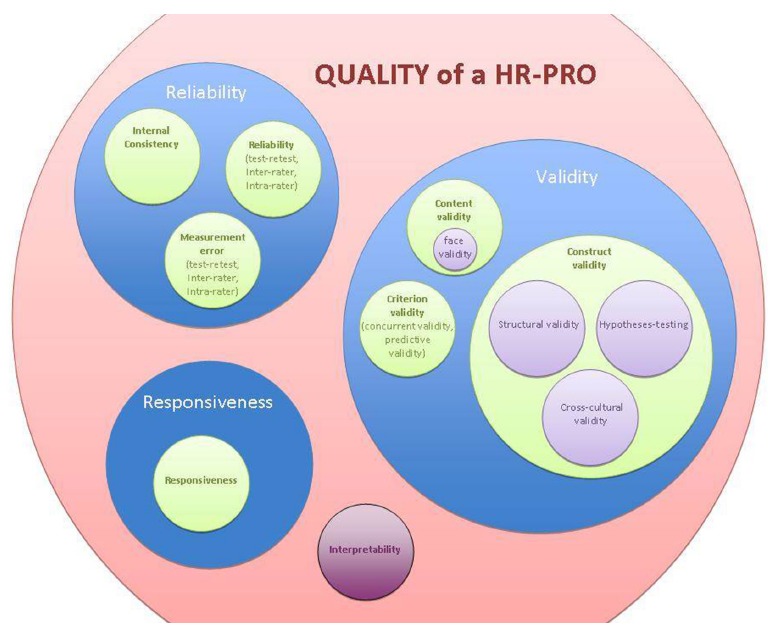
COSMIN taxonomy of relationships of measurement properties. COSMIN: COnsensus-based Standards for the selection of health Measurement INstruments; HR-PRO: health related-patient reported outcome.

We first developed a taxonomy and reached consensus on definitions of the measurement properties (see Table 1)^2^. Nine measurement properties clustered within three domains, i.e. reliability, validity and responsiveness, were considered relevant in the evaluation of outcome measurement instruments (Figure 1).

**Table 1.**
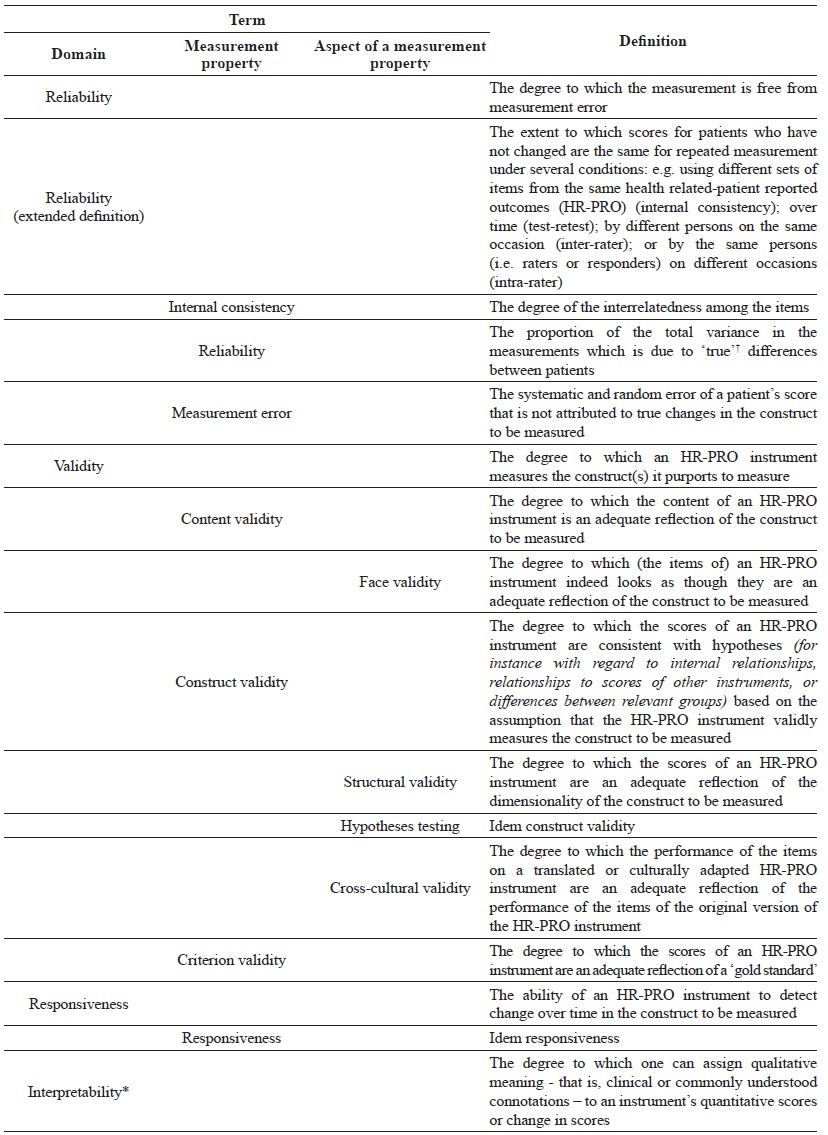
Definitions of domains, measurement properties, and aspects of measurement properties.

†The word 'true' must be seen in the context of the CTT, which states that any observation is composed of two components - a true score and error associated with the observation. 'True' is the average score that would be obtained if the scale were given an infinite number of times. It refers only to the consistency of the score, and not to its accuracy^15^. *Interpretability is not considered a measurement property, but an important characteristic of a measurement instrument.

### COSMIN checklist

We developed a critical appraisal tool, i.e. the COSMIN checklist^12^^-^ 14, containing standards for evaluating the methodological quality of studies on the measurement properties of outcome measurement instruments. The COSMIN checklist and a supplementary manual can be optained from the COSMIN website^16^. For each measurement property a box with standards was developed. These standards describe design requirements and preferred statistical methods. For example, in a high quality study of internal consistency, first a check for the unidimensionality of the (sub)scale should be done (Box A item 5 of the COSMIN checklist)^12^. Subsequently the internal consistency statistic should be calculated for the items of this unidimensional (sub)scale (Box A item 7)^12^. Other standards concern, for instance, using an appropriate time interval between test and retest administration when investigating test-retest reliability and measurement error (Box B and C item 8)^12^, or formulating a priori hypotheses for hypotheses testing (a form of construct validity) (Box F item 4)^12^.

When examining the interrater reliability and agreement of the items of the COSMIN checklist, we found that the reliability of the individual items was low (i.e. only 6% of the items had a Kappa statistic above 0.75), but that the agreement between raters was appropriate for 80% of the items^17^. When using the COSMIN checklist in a systematic review, we recommend getting some prior on-the-job training and experience, completing it by two independent raters, and reaching consensus about the ratings^17^. To use the COSMIN checklist in a systematic review of measurement instruments, we developed a four-point rating system for scoring the items of the COSMIN checklist^14^. With this version it is possible to calculate overall methodological quality scores per study on a measurement property. This is useful and enlightening in systematic reviews, as it allows to present conclusions on the quality of the instruments under study accompanied by various levels of evidence^14^.

### Search filter for finding studies on measurement properties

To facilitate the selection of outcome measurement instruments to be included in a systematic review of measurement instruments, a search filter was developed and validated in cooperation with clinical librarians for finding studies on measurement properties in PubMed^18^. In such a review, the filter can be combined with search terms for the outcome and the population of interest. The filter for finding studies on measurement properties showed to have a sensitivity of 97.4% and a positive predictive value of 4.4%. We translated this filter for EMBASE and CINAHL, and all filters are available from the COSMIN website^16^.

### Protocol for systematic reviews of outcome measurement instruments

Systematic reviews of outcome measurement instruments are important for the evidence-based selection of instruments. In such a review, the measurement properties of all outcome measurement instruments for a specific construct in a specific population are described and compared according to predefined criteria, and a conclusion is drawn about the most appropriate instrument.

We developed a protocol for performing systematic reviews of measurement instruments, including a 10-step procedure (available from the COSMIN website). In this protocol we describe how the COSMIN search filter^18^ can be used to identify all relevant outcome measurement instruments, as well as how the COSMIN checklist^12^ can be used to assess the quality of the included studies. In addition to the search filter for studies on measurement properties, and if the review concerns PROMs, a PROM filter developed by the University of Oxford can be used (available from the COSMIN website).

In addition, we describe the method of a best evidence synthesis in which the number of studies, their quality and (consistency of) results can be combined to determine the strength of the evidence for each measurement property. For example, strong evidence for a positive reliability is obtained when consistent positive results (ICCs or Kappas >0.70) are found in at least two studies of good quality or one study of excellent quality. The procedure is similar to the Grading of Recommendations Assessment, Development and Evaluation (GRADE) approach^19^ that is used in reviews of clinical trials. Previously developed cut-off values (such as ICC or Kappas >0.70) are used to determine whether an outcome measurement instrument has good measurement properties^20^.

In 2009 we concluded, based on a review of systematic reviews of measurement instruments, that the quality of these reviews should and could be improved^21^. Recently, we updated this review, and concluded that the quality of published systematic reviews of measurement instruments has improved^22^. However, there is still room for improvement with regards to the search strategy, and especially the quality assessment of the included studies and instruments as well as the data synthesis. Therefore, we are currently updating the protocol for performing systematic reviews of measurement instruments, aiming to publish it as a peer-reviewed guideline for systematic reviews of outcome measurement instruments (manuscript in preparation). In this way, we aim to contribute to the improvement of systematic reviews of measurement instruments.

### Database for systematic review of outcome measurement instruments

The COSMIN initiative maintains an overview of published systematic reviews of outcome measurement instruments. This overview is presented in a searchable database available on the COSMIN website^16^. Currently, it contains 569 systematic reviews and we aim to update this overview yearly. The COSMIN database provides a good starting point to search for and select outcome measurement instruments.

### Guideline for selecting outcome measurement instruments for outcomes included in a Core Outcome Set

COSMIN collaborated with the COMET (Core Outcome Measures in Effectiveness Trials) initiative to develop a guideline for the selection of outcome measurement instruments for outcomes included in a COS^10^. We reached consensus among a large group of experts on four main steps in the selection of outcome measurement instruments for COS: Step 1) conceptual considerations; Step 2) finding existing outcome measurement instruments; Step 3) quality assessment of outcome measurement instruments; and Step 4) generic recommendations on the selection of outcome measurement instruments for outcomes included in a COS. The resulting consensus-based guideline can be used by COS developers in defining *how* to measure core outcomes (submitted publication by Prinsen CA, et al. How to select outcome measurement instruments for outcomes included in a 'Core Outcome Set' - a practical guideline).

### Ongoing and future studies

At the moment, we work on updating the COSMIN checklist. Over the past years, users of the COSMIN checklist have identified gaps in the available standards. Recent regulatory guidelines on outcome measurement instruments development and evaluation call for an extension of the COSMIN checklist with respect to its standards for the quality of studies on content validity within the specific context of interest. Therefore, a Delphi study is underway which aims to reach consensus on new COSMIN standards and criteria for evaluating the content validity (including face validity) of PROMs. In these new standards, the quality of the development process of PROMs will be taken into account, and criteria for what constitutes good content validity will be developed.

In addition, a shift has taken place in recent years from the use of traditional statistical methods (i.e. Classical Test Theory (CTT)) to the recommended use of newer statistical methods (e.g. Item Response Theory (IRT)^23^ and Rasch Measurement Theory^24^) analyses for developing and evaluating outcome measurement instruments. This requires an extension of the COSMIN standards for studies using IRT and Rasch methods. Clear methodological advantages of using IRT or other modern test theory methods over or in addition to CTT have been described^25^. Well-developed IRT-based instruments, have probably better measurement properties than CTT-based instruments^26^^,^ 27. In addition, IRT allows for Computer Adaptive Testing (CAT), a method of questionnaire administration in which a computer algorithm iteratively selects questions based on previous answers. Questionnaires that are completed by CAT dramatically decreases the burden for patients to complete questionnaires and improving precision^28^^-^ 31. Examples of IRT-based instruments are the Patient Reported Outcomes Measurement Information System (PROMIS) instruments, which are available as CAT instruments as well as static short forms^32^. A next step to be addressed is to achieve consensus among an international group of experts on standards for the methodological quality of studies using IRT and Rasch methods for evaluating measurement properties and to operationalize these standards into a user-friendly and easily applicable checklist to be used e.g. in systematic reviews of outcome measurement instruments.

The COSMIN standards were originally developed for evaluating the quality of studies on the measurement properties of PROMs. Although the COSMIN standards have also been used in systematic reviews of other types of outcome measurement instruments, adaptations are required to use the COSMIN standards for evaluating the quality of studies on the measurement properties of other patient-centered outcome measurement instruments, such as clinician-reported outcome measure (e.g. a goniometer to measure range of motion), or a performance based test (e.g. a six minute walk test to measure walking speed). It is our ambition to develop new standards specific for other types of instruments.

Finally, we want to develop reporting guidelines for studies on measurement properties, and for systematic reviews on measurement properties.

### Need for high quality systematic reviews of outcome measurement instruments and Core Outcome Set development

By the development of the COSMIN tools described above and by generating awareness for the importance of selecting high quality instruments, COSMIN aims to accomplish that researchers and clinicians make their choices on outcomes and outcome measurement instruments more informed. We plea for more standardization in the use of outcomes and outcome measurement instruments. We support the aim of the COMET initiative to stimulate the development of COS. The use of COS will lead to more standardization in outcome reporting in specific areas of research, making it easier for the results of trials to be compared and combined as appropriate. COSMIN strongly encourages researchers to perform high quality systematic reviews of outcome measurement instruments. More high quality systematic reviews of outcome measurement instruments are needed to make an informed choice for the best instrument for a specific purpose and for stopping the use of poor outcome measurement instruments.
